# Bortezomib treatment induces a higher mortality rate in lupus model mice with a higher disease activity

**DOI:** 10.1186/s13075-017-1397-7

**Published:** 2017-08-11

**Authors:** Tomoko Ikeda, Hiroshi Fujii, Masato Nose, Yukiko Kamogawa, Tsuyoshi Shirai, Yuko Shirota, Tomonori Ishii, Hideo Harigae

**Affiliations:** 1 Department of Hematology and Rheumatology, Tohoku University Graduate School of Medicine, 1-1 Seiryo-machi, Aoba-ku, Sendai, Miyagi 980-8574 Japan; 2 Institue for Promotion of Advanced Science and Technology, Ehime University, Matsuyama, Japan

**Keywords:** Systemic lupus erythematosus, Proteasome inhibitor, Model animal

## Abstract

**Background:**

Bortezomib (Bz) is a proteasome inhibitor that directly targets antibody-producing plasma cells. We recently reported the first randomized control trial that evaluated the effects of Bz in patients with systemic lupus erythematosus (SLE). In that study, we demonstrated that Bz treatment is associated with many adverse reactions in patients with refractory disease. In the present study, we examine the therapeutic and toxic effects of Bz on MRL/MpJ-*lpr/lpr* (MRL/lpr) mice with severe disease activity.

**Methods:**

Female MRL/lpr mice at 10 and 14 weeks of age were treated with phosphate buffered saline (PBS) (*n* = 19), Bz (750 μg/kg twice weekly) (*n* = 27), or cyclophosphamide (Cyc) (1 mg/body, once in 2 weeks) (*n* = 20). Cellular subsets, serum immunoglobulin, anti-double-stranded DNA (anti-dsDNA) antibody titer, and a pathological index of glomerulonephritis were then analyzed at 22 weeks of age. Survival curves of the 10-week-old and 14-week-old Bz-treated groups were compared. Blood counts, creatinine, liver enzymes, and serum cytokine levels were measured 1 week after Bz treatment. Gene expression profiling of spleens from Bz and Cyc treatment mice were compared with those from control mice.

**Results:**

The anti-dsDNA antibody levels were significantly higher in 14-week-old than in 10-week-old mice, indicating a higher disease activity at 14 weeks. A significant decrease in the number of splenic cells and glomerulonephritis index was observed in Bz-treated and Cyc-treated mice. Bz, but not Cyc, significantly decreased serum immunoglobulin and anti-dsDNA antibody titer levels. Survival curve analysis revealed a significantly higher mortality rate in 14-week-old than in 10-week-old Bz-treated and control groups. Following two injections of Bz, serum IL-6 and TNF-α levels were significantly more elevated in 14-week-old than in 10-week-old mice. Potentially immunogenic molecules, such as heat shock proteins, were characteristically upregulated in spleens of Bz-treated but not Cyc-treated mice.

**Conclusions:**

In spite of its therapeutic effect, Bz treatment had more toxic effects associated with increased proinflammatory cytokine levels in mice with a higher disease activity. Understanding the mechanism of the toxicity and developing preventive strategies against it is important for the safe clinical application of Bz in human SLE.

**Electronic supplementary material:**

The online version of this article (doi:10.1186/s13075-017-1397-7) contains supplementary material, which is available to authorized users.

## Background

In systemic lupus erythematosus (SLE), autoreactive antibodies, such as anti-dsDNA antibodies, are considered to trigger tissue inflammation [1]. One therapeutic approach is to diminish the production of pathogenic antibodies. Toward this goal, several experimental therapeutic strategies have targeted molecules involved in pathways that control B-cell activation into plasma cells [2, 3]. It is noteworthy that some SLE patients are refractory to conventional therapies, and thus require high doses of steroids, which are associated with many side effects.

Bortezomib (Bz) is a proteasome inhibitor that enhances the accumulation of unfolded proteins with the induction of ER stress, leading to apoptotic cell death [4]. Plasma cells that synthesize high levels of immunoglobulins have relatively high sensitivity to Bz [5], which partly explains the clinical success of Bz in treating multiple myeloma [6]. Bz has been reported to inhibit the production of anti-dsDNA antibody and improve glomerulonephritis, proteinuria, and survival of lupus model mice, such as NZB/W [7–12] and MRL/lpr [7, 13, 14] mice. Recently, Bz treatment has been reported to be effective in treating human SLE [15, 16]. Our group was the first to report a randomized control study on the effects of Bz in patients with active lupus [17]. In that study, all patients with Bz treatment developed fever within 1 week of Bz administration. In order to safely use Bz to treat patients with refractory SLE, we need to clarify its mechanisms of toxicity in order to devise preventive strategies to mitigate its adverse effects.

MRL/lpr mice bearing *Fas* mutation on the MRL background spontaneously develop pathological lesions, such as glomerulonephritis, systemic vasculitis, arthritis, and sialoadenitis, as well as splenomegaly and lymphadenopathy [18]. Glomerulonephritis is characterized by endocapillary proliferative and wire loop lesions, which resemble lupus nephritis. MRL/lpr mice also have deposition of immunoglobulins in tissue lesions, especially in glomeruli, associated with serological abnormalities, such as increased anti-dsDNA antibodies and anti-Sm antibodies [19], rheumatoid factor [20], IgG3 [21], and skewed Th1 cytokine levels [22, 23]. Because the inoculation of hybridoma clones generated from splenic B cells from MRL/lpr mice into SCID mice could induce various glomerular lesions involving a endocapillary proliferative and a wire loop type [24–27], MRL/lpr mice are considered suitable models for antibody-mediated diseases and thus have been used to elucidate the efficacy of various drugs for SLE [28–32].

In the present study, we demonstrated that immunological activity was higher in 14-week-old than 10-week-old MRL/lpr mice, that Bz treatment was associated with a higher mortality rate in 14-week-old than in 10-week-old MRL/lpr mice, and that, in spite of its therapeutic effects on antibody production, Bz treatment causes significantly elevated serum levels of IL-6 and TNF-α in 14-week-old MRL/lpr mice.

## Methods

### Mice and drug administration

Eight-week-old female MRL/lpr mice were purchased from Jackson Laboratories (Bar Harbor, ME, USA). The mice were housed and maintained in the animal facilities of Tohoku University School of Medicine. Bz was purchased from Santa Cruz (Dallas, TX, USA) and prepared at a concentration of 10 mg/ml. Cyclophosphamide (Cyc) was purchased from Sigma-Aldrich (St. Louis, MO, USA) and prepared at a concentration of 5 mg/ml. We injected the mice subcutaneously with Bz (750 μg/kg body) twice weekly or intraperitoneally with Cyc (1 mg/body = 30–35 mg/kg) once in 2 weeks at the ages indicated. Control mice were injected subcutaneously or intraperitoneally with the same volume of phosphate buffered saline (PBS) instead of Bz and Cyc, respectively. The mice were euthanized by cervical dislocation at the specified ages, and samples were analyzed for immunological activities and to assess the effects and toxicities of drugs. The study protocols were reviewed by the Institutional Laboratory Animal Care and Use Committee of Tohoku University, and finally approved by the President of University.

### Flow cytometry

Bone marrow, spleen, and axillary lymph nodes were minced in Hank’s balanced solution and filtered through a 70-μm cell strainer (Falcon, Tewksbury, MA, USA) to prepare single-cell suspensions. After red blood cell (RBC) lysis with 1× RBC lysis buffer (eBioscience, San Diego, CA, USA), the cells were first stained with the following antibodies for 20 min on ice: PE-anti-Thy1.2 (clone 53-2.1; BD Biosciences, San Jose, CA, USA), PerCP-Cy5.5-anti-CD4 (clone RM4-5; BD Biosciences), APC-anti-CD138 (clone 281-2; BD Biosciences), PE-Cy7-anti-CD8 (clone 53-6.7; BD Biosciences), APC-Cy7-anti-CD19 (clone 6D5; BD Biosciences), and ViroGreen-anti-B220 (clone RA3-6B2; Miltenyi, Teterow, Germany). After washing with PBS containing 1% bovine serum albumin (BSA; Sigma-Aldrich), the cells were fixed with Cytofix/Cytoperm Fixation (BD Biosciences) and permeated with permeabilization solution (BD Biosciences). Second, the cells were stained intracellularly with Horizon450-anti-IgL-κ antibody for 30 min. Flow cytometric analysis was performed using FACSCanto II with FACS Diva (BD Biosciences). Acquired data were analyzed with FlowJo software (Tree Star Inc., Ashland, OR, USA). Absolute cell numbers were calculated based on the frequency of the cell population and the total cell number of each organ. Each cell population was defined as follows: B cells as CD19^+^, plasma cells as CD138^+^IgL-κ^+^, CD4 T cells as CD4^+^CD8^–^Thy1.2^+^, CD8 T cells as CD4^–^CD8^+^Thy1.2^+^, and lpr T cells as CD4^–^CD8^–^Th1.2^+^B220^+^.

### Serum immunoglobulin subclasses and anti-dsDNA antibody titer

For the study, 96-well ELISA plates (Costar, Corning, NY, USA) were coated overnight with goat anti-mouse Ig antibody (SouthernBiotech, Birmingham, AL, USA) or with calf thymus DNA (20 μg/ml) treated with S1 nuclease (Sigma). After washing three times with PBS containing 0.1% Tween 20, ELISA plates were blocked with 100 μl of Syn Block (ImmunoChemistry, Bloomington, MN, USA) for 2 h at room temperature. Mouse serum diluted with PBS containing 1% BSA and 0.1% Tween 20 was added to the plates at 50 μl/well and incubated for 1.5 h at room temperature. After washing three times, each immunoglobulin-specific or anti-dsDNA antibody-specific antibody was detected with horseradish peroxidase-anti-mouse IgG1, IgG2a, IgG2b, IgG3, IgM (Abcam, Cambridge, UK), or IgG (Rockland, Limerick, PA, USA) antibody and TMB liquid (Sigma). The plates were analyzed with ELISA plate reader iMARK (Bio-Rad, CA, USA). Optical densities for anti-dsDNA antibody were converted to arbitrary units based on the reference serum of 38-week-old MRL/lpr mice. Optical densities for each immunoglobulin subclass were converted to milligrams per deciliter based on the reference mouse serum with known concentrations of each immunoglobulin (Bethyl, Montgomery, TX, USA).

### Histopathological analysis of glomerulonephritis

Kidney samples obtained from the mice were fixed with 10% formalin and embedded in paraffin. The samples were stained with hematoxylin and eosin. Histopathological examinations for glomerulonephritis are described elsewhere [33]. The following criteria were used for grading each glomerulus: grade 0, no recognizable lesion in a glomerulus; grade 1, mild cell proliferation and/or cell infiltration; grade 2, the same as grade 1 with mesangial proliferation, lobulation, and hyaline droplet; and grade 3, the same as grade 2 with crescent and granuloma formation and/or hyalinosis. The reader, who was blinded to whether the specimens were from control or drug-treated mice, obtained 20 glomeruli from one mouse. The glomerulonephritis index of an individual mouse was expressed as the mean of the grading of 20 glomeruli. For immunofluorescence, kidneys were snap-frozen in OCT compound (Sakura Finetek, USA) before sectioning. Cryostat sections were stained with FITC-goat anti mouse IgG antibody and observed on a BZ-8000 T fluorescent microscope (Keyence, Tokyo, Japan).

### Complete blood count and serum biochemical analysis

Complete blood count was performed using Microsemi LC-662 (Horiba-seisakusho, Tokushma, Japan). Serum creatinine (Cr), aspartate aminotransferase (AST), alanine aminotransferase (ALT), uric acid, calcium, and phosphate levels were quantified using standard laboratory methods (Nagahama Life Science Laboratory, Shiga, Japan).

### Cytokines

Serum concentrations of IFN-γ, IL-6, and TNF-α were measured with a cytokine Cytometric Beads Array (BD Biosciences) following the manufacturer’s instruction. The concentration of each cytokine was calculated with FCAP Array Version 3.0 (BD Biosciences).

### Microarray

Fourteen-week-old MRL/lpr mice were injected with Bz (750 μg/kg subcutaneously), Cyc (1 mg/body intraperitoneally), or PBS. The mice were sacrificed, and the spleens were removed 6 h after injecting the drugs. RNA was extracted from the spleen samples using RNAiso Plus (TaKaRa Biotech, Shiga, Japan) and cleaned up with the RNeasy MiniElute Cleanup kit (QIAGEN) according to the manufacturer’s instructions. Cyanine-3-labeled cRNA was prepared from 0.1 μg total RNA using the Low Input Quick Amp Labeling Kit (Agilent Technologies, Santa Clara, CA, USA) and hybridized to a SurePrint G3 Mouse GE 8 × 60 K Microarray (Agilent Technologies) according to the manufacturer’s instructions. Gene expression profiling of Bz-treated and Cyc-treated mice were compared with control mice using Gene Spring software (Agilent Technologies). Genes that significantly increased (>2-fold) or decreased (<0.5-fold) in expression were considered for analysis. The genes with significantly changed expressions were studied with Ingenuity Pathway Analysis (IPA) for canonical pathways.

### In-vitro induction of *HSPA1A* and *HSPB1*

Cell suspensions were prepared from axillary lymph nodes harvested from 10-week-old and 14-week-old female MRL/lpr mice. Lymph node cells were seeded in 24-well plates at a density of 1.0 × 10^6^/well. The cells were then treated with Bz (100 μM) and staurosporine (100 μM) (Sigma) in RPMI 1640 (Cellgro) supplemented with 10% FCS, penicillin (100 IU/ml), and streptomycin (100 μg/ml). After 6 hours of incubation, total RNA was extracted using Trizol (Invitrogen, Carlsbad, CA, USA) and cDNA was synthesized using the ReverTra Ace qPCR RT Kit (Toyobo, Tokyo, Japan). PCR was performed with 5 μl of cDNA sample and 15 μl of PCR solution from the Quantifect SYBR Green PCR Kit (QIAGEN), containing 200 nM of sense and anti-sense primers, respectively. The primer sequences were as follows: *GAPDH*: sense 5′-TGCCCCCATGTTTGTGATG-3′ and anti-sense 5′-TGTGGTCATGAGCCCTTCC-3′; *HSPA1A1* (HSP70): sense 5′-TGTTCGAGGGCATCGACTTCT-3′ and anti-sense 5′-TGATGCTCTTGTTCAGGTCGC-3′; and *HSPB1* (HSP27): sense 5′-CACTGGCAAGCACGAAGAAAG-3′ and anti-sense 5′-GGCCTCGAAAGTAACCGGAAT-3′.

Using a CFX96 Real-Time Detection System (BioRad, Hercules, CA, USA), PCR mixtures were incubated for 15 min at 95 °C, and then amplified for 40 cycles for 10 sec at 95 °C, 30 sec at 60 °C, and 30 sec at 72 °C. Gene copy numbers were calculated by comparing Ct values of each sample with a standard curve, which was generated by amplifying serially diluted plasmid samples containing the relevant sequences.

### Statistical analysis

For calculation of statistical significance, the paired Student’s *t* test, nonparametric Mann–Whitney test, or one-way ANOVA analyses with Turkey post-hoc test was used as indicated. Statistical analysis was performed with Graphpad Prism 7 software. *p* < 0.05 was considered statistically significant. Results were depicted as median with quartile range or mean value ± SEM, as indicated.

## Results

### Comparison of 10-week-old and 14-week-old MRL/lpr mice

**Fig. 1:**
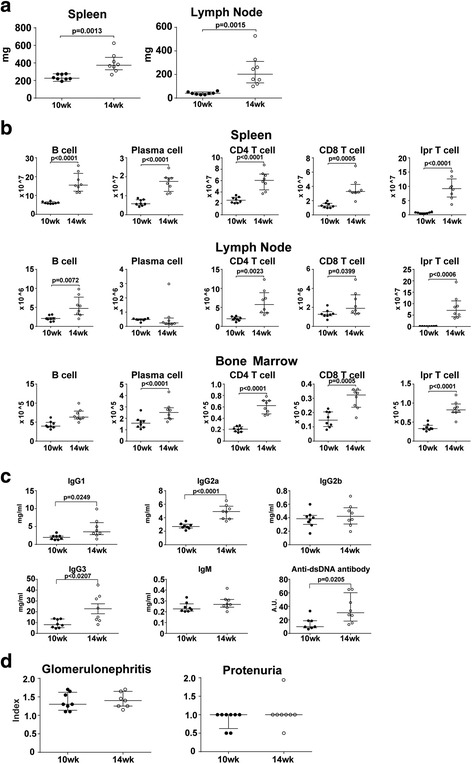
Immunological profiles of MRL/lpr mice at 10 and 14 weeks of age. After the mice were sacrificed, the spleens, axillary lymph nodes, femurs, and kidneys were removed and analyzed. **a** Weight of spleen and axillary lymph nodes. **b** Number of cellular subsets of spleen, axillary lymph nodes, and bone marrow from one femur. **c** Serum concentration of immunoglobulin subsets and anti-dsDNA antibody titer. **d** Histopathological analysis of glomerulonephritis at 10 and 14 weeks of age. Values expressed as median with quartile range. Statistical analysis performed using the nonparametric Mann–Whitney test

We tried to examine the effects of Bz on MRL/lpr mice with higher disease activity. First, to determine the age at which the mice developed early or advanced stage disease, we compared the disease activity of MRL/lpr mice at 10 and 14 weeks of age. As shown in Fig. 1a, the spleens and lymph nodes of 14-week-old mice were heavier than those of the 10-week-old mice. Flow cytometric analysis revealed significantly more B cells, plasma cells, CD4 T cells, CD8 T cells, and lpr T cells in the spleens, lymph nodes, and bone marrow of 14-week-old than 10-week-old mice. However, the lymph node plasma cells were identical (Fig. 1b). Among these cell populations, lpr T cells showed remarkable accumulation into spleens (14.5-fold) and lymph nodes (92.6-fold) of 14-week-old compared with 10-week-old mice. Serological analysis revealed that serum levels of IgG1, IgG2a, IgG3, and anti-dsDNA antibody were significantly higher in the 14-week-old than in the 10-week-old mice. IgG2b and IgM were, however, not significantly different between the groups (Fig. 1c). In contrast to serological and cytological analysis, a pathological index for glomerulonephritis and proteinuria were not significantly different between the 10-week-old and 14-week-old mice (Fig. 1d). These data suggest that MRL/lpr mice at 14 weeks of age showed more immunological activity than those at 10 weeks of age.

### Therapeutic effect of Bz on 14-week-old MRL/lpr mice

**Fig. 2:**
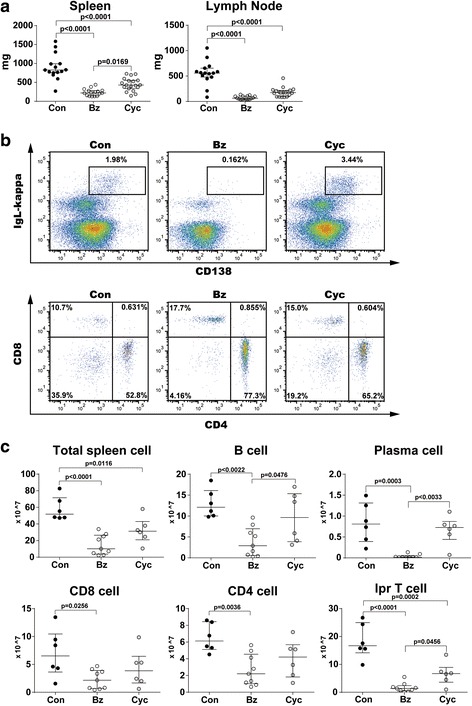
Therapeutic effects of Bz and Cyc on cellular subsets of 14-week-old and 22-week-old MRL/lpr mice. MRL/lpr mice aged 14 weeks were injected subcutaneously with Bz (750 μg/kg, twice weekly) or intraperitoneally with Cyc (1 mg/body weight, once in 2 weeks). At 22 weeks of age, mice were sacrificed and analyzed. **a** Weight of spleens and axillary lymph nodes. **b** Representative data for flow-cytometric analysis of splenic plasma cells (IgL-κ^+^CD138^+^) and T-cell subsets among Thy1.2^+^ T cells in Bz-treated or Cyc-treated mice. **c** Absolute numbers of each cellular subset in spleens. Values expressed as median with quartile range. Statistical analysis performed using one-way ANOVA analyses with Turkey post-hoc test. *Bz* bortezomib, *Con* control, *Cyc* cyclophosphamide

**Fig. 3:**
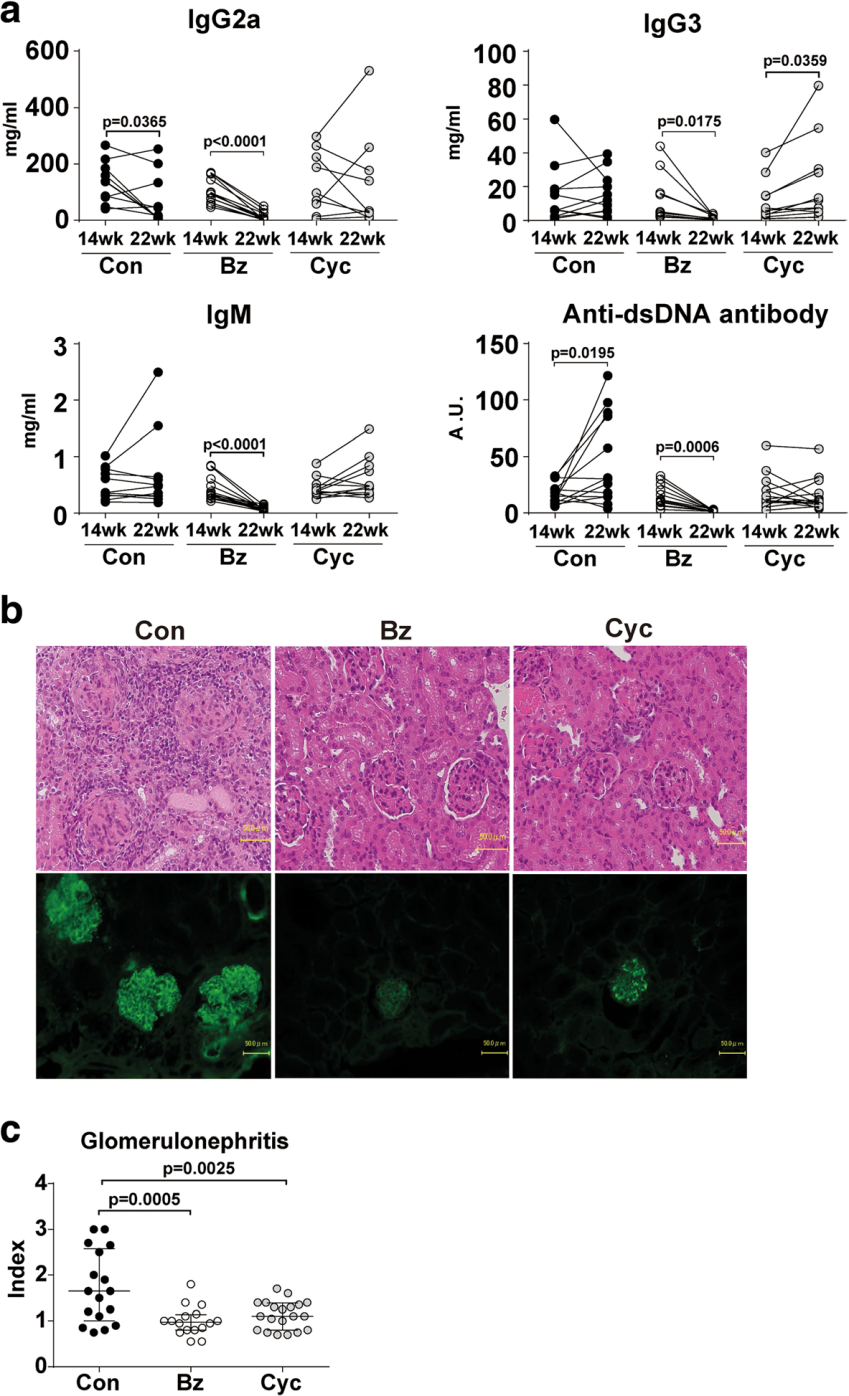
Therapeutic effects of Bz and Cyc on serum and renal histopathology of 14-week-old and 22-week-old MRL/lpr mice. **a** Change of serum immunoglobulin subclasses and titer of anti-dsDNA antibody after Bz or Cyc treatment. **b** Representative histopathology of glomerulonephritis: (*upper panel*) hematoxylin–eosin staining (×100) and (*lower panel*) immunofluorescent analysis of glomerular lesions stained with FITC-anti IgG antibody. Cellular infiltrates and glomerular deposition of IgG were reduced in Bz-treated and Cyc-treated mice compared with controls. **c** Histopathological analysis of glomerulonephritis after Bz or Cyc treatment. Values expressed as median with quartile range. Statistical analysis performed using paired *t* test (**a**) and one-way ANOVA analyses with Turkey post-hoc test (**c**). In the control group, 19 mice aged 14 weeks were treated and 17 of them survived at 22 weeks of age (two mice died). In the Bz group, 27 mice aged 14 weeks were treated but only 16 mice survived at 22 weeks of age (11 mice died). However, in the Cyc group, 20 mice aged 14 weeks were treated and all survived at 22 weeks of age (no mice died). *anti-dsDNA* anti-double-stranded DNA, *A.U.* arbitrary units, *Bz* bortezomib, *Con* control, *Cyc* cyclophosphamide

To examine the therapeutic effect of Bz on serologically active mice, we compared 22-week-old MRL/lpr mice treated with Bz and Cyc from 14 weeks of age, at 2 days after the last injection of Bz and Cyc. Both Bz and Cyc treatments significantly reduced the splenomegaly and lymphadenopathy in MRL/lpr mice compared with the control mice (Fig. 2a). In spleens, the frequencies of plasma cells were decreased in Bz-treated mice and the frequencies of lpr T cells among Thy1.2^+^ T cells were reduced in both Bz-treated and Cyc-treated mice (Fig. 2b). Bz significantly decreased the number of B cells, plasma cells, CD4 T cells, CD8 T cells, and lpr T cells in MRL/lpr mice compared with the control mice (Fig. 2c). Among these cell subsets, plasma cells and lpr T cells were drastically reduced by Bz treatment (4.24% and 9.25% of control, respectively). Cyc also more significantly decreased the number of lpr T cells in MRL/lpr mice than in control mice (Fig. 2c). These results indicate that plasma cells and lpr T cells have higher sensitivities to Bz treatment than the other cellular subsets. Serum levels of Ig subclasses and anti-dsDNA antibody were markedly reduced by Bz treatment. On the other hand, Cyc treatment showed changes in the serological level of anti-dsDNA antibody and Ig subclasses in a variable manner but did not induce a significant decrease in either of them (Fig. 3a). Bz and Cyc treatment markedly decreased cellular invasion in or around the glomeruli and diminished the glomerular deposition of immunoglobulins (Fig. 3b). These findings were associated with improved histopathological indexes of glomerulonephritis (Fig. 3c). Taken together, administration of Bz to 14-week-old MRL/lpr mice attenuated the serological and pathological disease activity at 22 weeks of age. Although Bz treatment for 14-week-old mice (*n* = 27) suppressed their immunological activity, 11 mice unexpectedly died within 22 weeks of age.

### Survival curve

**Fig. 4:**
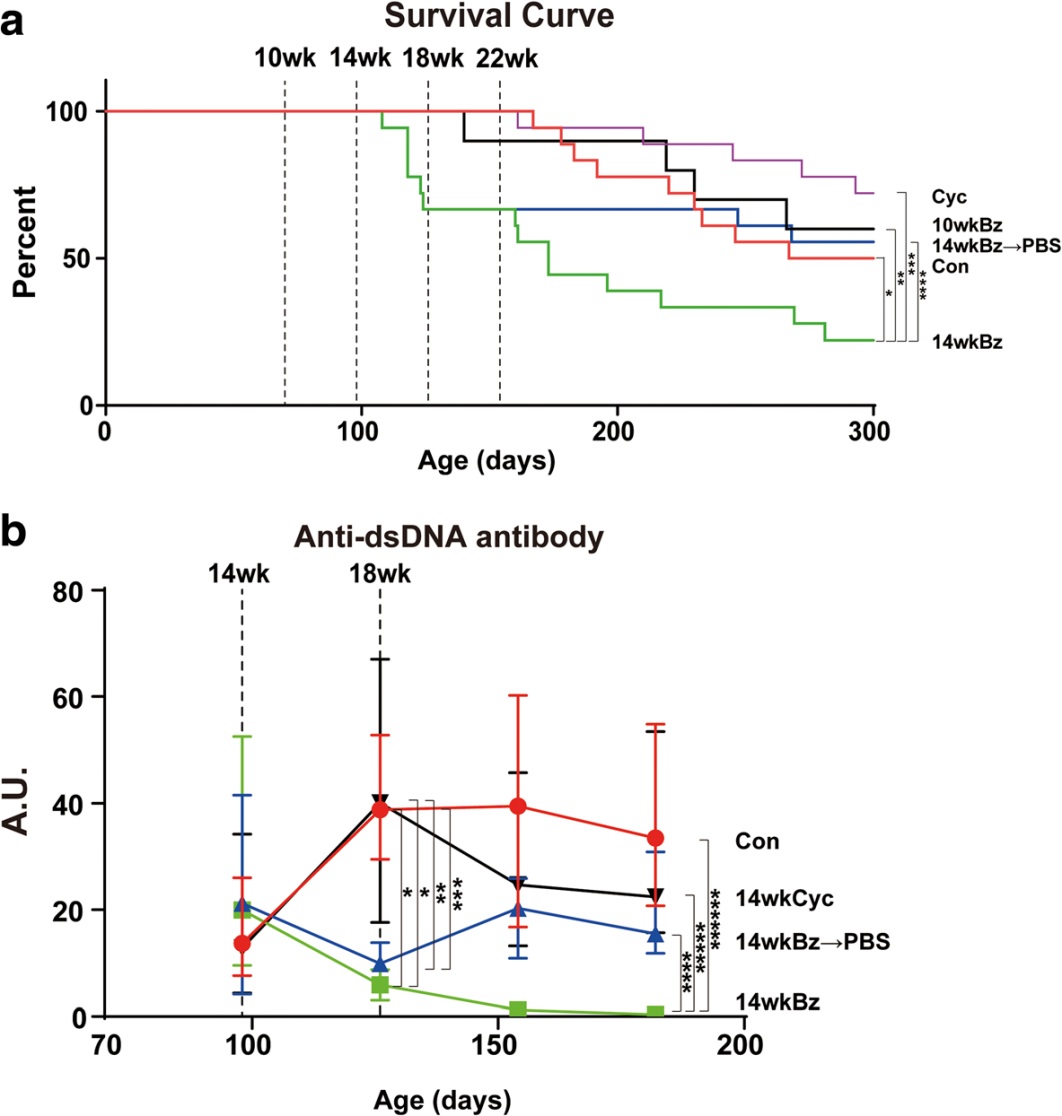
Kaplan–Meier survival analyses of MRL/lpr mice and anti-dsDNA antibody titer as a function of age. **a** Kaplan–Meier survival curve. Experimental groups of mice treated with Bz or Cyc: control (*Con*, *red*) (*n* = 18), injected with vehicle beginning at 10 weeks of age; *10wkBz* (*black*) (*n* = 10), injected with Bz twice in a week beginning at 10 weeks of age; *14wkBz* (*green*) (*n* = 18), injected with Bz twice a week beginning at 14 weeks of age; *14wkBz → PBS* (*blue*) (*n* = 18), injected with Bz twice a week beginning at 14 weeks until 18 weeks of age, PBS then injected from 19 weeks onward; *Cyc* (*purple*) (*n* = 18), injected with Cyc once in 2 weeks. Log-rank test: *p = 0.0245, **p = 0.032, ***p = 0.0005, ****p = 0.010 (after 22 weeks of age). **b** Anti-dsDNA antibody titer in the course of treatment with Bz. Blood was drawn from the tail veins at 14, 18, 22, and 26 weeks of age. Sera were analyzed for anti-dsDNA antibody: control (*Con*, *red*) (*n* = 18), injected with vehicle beginning at 10 weeks of age; *14wkBz* (*green*) (*n* = 18), injected with Bz twice a week beginning at 14 weeks of age; *14wkBz → PBS* (*blue*) (*n* = 18), injected with Bz twice a week beginning at 14 weeks of age until 18 weeks of age, PBS then injected from 19 weeks onward; *Cyc* (*black*) (*n* = 18), injected with Cyc once in 2 weeks. Values expressed as median with quartile range. Statistical analysis performed using one-way ANOVA analyses with Turkey post-hoc test. *p < 0.001, **p =0.0012, ***p =0.0032, ****p =0.00272, *****p =0.00075, ******p =0.00064. *anti-dsDNA* anti-double-stranded DNA, *A.U.* arbitrary units, *Bz* bortezomib, *Cyc* cyclophosphamide (Color figure online)

Next, to examine whether the lethal toxic effects of Bz depend on the disease activity, we compared the survival curve of mice treated with Bz from 10 weeks or 14 weeks of age. Within 4 weeks after the initiation of Bz treatment, 33% of the mice died (Fig. 4a). Kaplan–Meier survival curve analysis revealed that Bz treatment from 14 weeks old significantly decreased the survival rate of mice, compared with control, Bz treatment from 10 weeks old, and Cyc treatment from 14 weeks old. In spite of the therapeutic effects shown in Figs. 2 and 3, Bz treatment exerted lethal toxic effects on immunologically active mice and not on less active younger mice. The mice survival rate, after discontinuing Bz treatment (14wkBz → PBS), significantly improved (Fig. 4a) even comparable with control mice, although anti-dsDNA antibody levels increased again after the discontinuation of Bz treatment at 18 weeks of age (Fig. 4b).

### Early toxic effects of Bz within 1 week

**Fig. 5:**
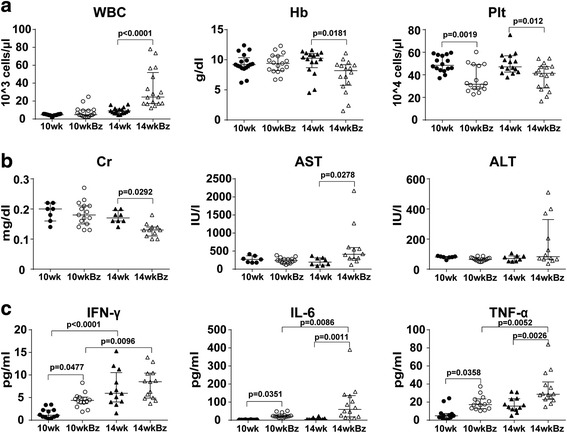
Early-phase toxic effects after Bz treatment. Mice aged 10 or 14 weeks were injected with vehicle or Bz twice on day 1 and day 4 (*10wk*, *14wk*, *10wkBz*, and *14wkBz*, respectively). One day after the second Bz injection, mice were sacrificed and blood specimens were analyzed. **a** Blood cell counts. **b** Renal and liver function tests: Serum Cr, AST, and ALT. **c** Serum IFN-γ, IL-6, and TNF-α levels. Values expressed as median with quartile range. Statistical analysis performed using one-way ANOVA analyses with Turkey post-hoc test. *ALT* alanine aminotransferase, *AST* aspartate aminotransferase, *Cr* creatinine, *Hb* hemoglobin, *IFN-*γ interferon-gamma, *IL-6* interleukin-6, *Plt* platelet, *TNF-*α tumor necrosis alpha, *WBC* white blood cell

We assumed that Bz exerted its lethal toxic effects early on 14-week-old mice after the initiation of treatment. Therefore, blood samples from mice treated twice with Bz at 10 or 14 weeks of age were analyzed (10wkBz and 14wkBz, respectively). White blood cells were increased, whereas hemoglobin levels were significantly decreased after Bz treatment of 14-week-old mice (Fig. 5a). However, platelets were significantly diminished by Bz treatment in both 10-week-old and 14-week-old mice (Fig. 5a). Serum AST was significantly elevated after Bz treatment of 14-week-old mice (Fig. 5b). Serum levels of IFN-γ were significantly higher in 14-week-old than in 10-week-old mice (Fig. 5c), mirroring the increased immunological activity. Bz treatment of mice at both 10 and 14 weeks of age significantly elevated serum IL-6 and TNF-α levels, but IFN-γ levels were not significantly changed by Bz treatment at 14 weeks of age. The increase in serum IL-6 and TNF-α levels was significantly higher in 14wkBz than in 10wkBz mice (Fig. 5c). These data suggest that Bz treatment at 14 weeks of age induced more inflammatory cytokine release than treatment at 10 weeks of age.

### Late toxic effects of Bz

**Fig. 6:**
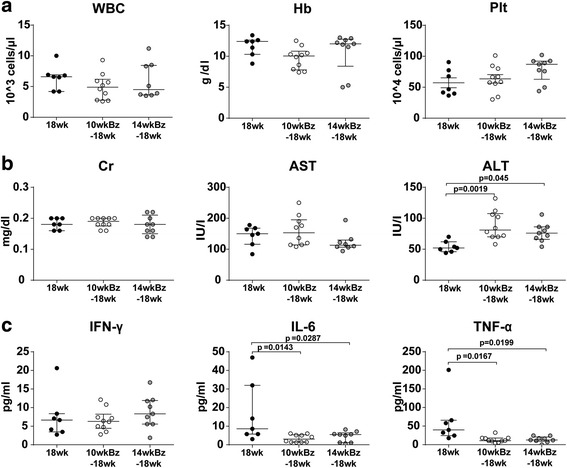
Late-phase toxic effects of Bz after treatment. Mice were injected with vehicle (*18wk*) or Bz twice a week beginning at 10 weeks (*10wkBz-18wk*) and 14 weeks (*14wkBz–18wk*) of age until 18 weeks of age. At 18 weeks of age, mice were sacrificed and the blood samples were analyzed. **a** Blood count analysis. **b** Renal and liver function test: Serum Cr, AST, and ALT. **c** Serum IFN-γ, IL-6, and TNF-α levels. Values expressed as median with quartile range. Statistical analysis performed using one-way ANOVA analyses with Turkey post-hoc test. *ALT* alanine aminotransferase, *AST* aspartate aminotransferase, *Cr* creatinine, *Hb* hemoglobin, *IFN-*γ interferon-gamma, *IL-6* interleukin-6, *Plt* platelet, *TNF-*α tumor necrosis alpha, *WBC* white blood cell

Next, to examine the toxic effects of long-term treatment with Bz, MRL/lpr mice were treated from 10 or 14 weeks of age, and blood analysis was performed at 18 weeks of age. In this experiment, five of 14 mice started on Bz at 14 weeks of age died before 18 weeks, which is consistent with the data in Fig. 3a. None of the controls and mice treated at 10 weeks of age died during this period. At 18 weeks of age, Bz treatment from 10 weeks (10wkBz-18wk) and 14 weeks (14wkBz-18wk) did not show a significant difference in blood counts (Fig. 6a) and Cr and AST levels (Fig. 6b). ALT was slightly increased, whereas both IL-6 and TNF-α were significantly decreased by Bz treatment (Fig. 6b, c).

### Comparison of gene expression profiling in spleens from Bz-treated and Cyc-treated mice

To elucidate the differences in the effects of Bz and Cyc on mice, gene expression profiles of spleens were compared using microarray analysis. Six hours after Bz or Cyc treatment, 74 genes with >2-fold increase and 102 genes with <0.5-fold decrease in Bz-treated mice and 49 genes with >2-fold increase and three genes with <0.5-fold decrease in Cyc-treated mice were uncovered (Table 1, Additional file 1). No common genes were significantly changed in both Bz-treated and Cyc-treated mice. The 70-kDa heat shock protein 1A (HSPA1A) was remarkably increased in Bz-treated mice (100.3-fold) (Table 1). Pathway analysis performed with IPA software revealed that upregulation and downregulation of some genes were significantly associated with distinct canonical pathways, supporting the different mechanisms of each drug (Additional file 2). The protein ubiquitin pathway was most significantly associated with Bz treatment (*p* = 0.000692) (Additional file 2), whereas the growth arrest and DNA damage inducible 45 (GADD45) and DNA damage-induced 14-3-3 signaling pathways were most significantly associated with Cyc treatment (*p* = 0.000575) (Additional file 2). The expression of some genes of the HSP family and proteasome subunit proteins was significantly increased in the protein ubiquitin pathway, whereas that of the genes of cyclin E2 (*CCNE2*) and cyclin dependent kinase 1 (*CDK1*) was significantly increased in the GADD45 and DNA damage-induced 14-3-3 signaling pathways (Additional file 3).

**Table 1:** Top 20 genes upregulated in the spleen by bortezomib or cyclophosphamide treatment relative to control mice

Bortezomib		Cyclophosphamide	
Symbol	Fold change	Symbol	Fold change
*HSPA1A*	100.3	*Dynap*	17.7
*UCHL1*	38.3	*Akr1c20*	8.74
*RNF207*	23.2	*CCSER1*	6.63
*TRIB3*	21.2	*AURKB*	6.50
*HSPB1*	20.6	*Olfr123*	6.38
*GDF15*	14.9	*ARHGAP19*	6.13
*CHAC1*	9.32	*NXF3*	6.01
*AFP*	7.44	*LRRC72*	5.85
*TRIM6*	6.81	*Cmtm2a*	5.83
*STMN4*	6.36	*CDK1*	5.79
*AOX1*	6.12	*LIPC*	5.04
*HSPB8*	5.93	*ASIC3*	4.51
*MAP1A*	5.77	*CCNE2*	4.434
*SRXN1*	5.56	*Serpinb6d*	4.21
*COMP*	5.55	*Gm9372*	3.84
*DNAH10*	5.42	*CEACAM20*	3.63
*CALR3*	5.36	*Gm10753*	3.60
*DYNC1I1*	5.04	*SPRY4*	3.54
*ATF3*	4.95	*EPB41L4B*	3.43
*KIF1A*	4.93	*Gm5093*	3.40

### In-vitro induction of heat shock protein gene expression in Bz-treated lymph node cells

As shown in the gene list presented in Table 1, *HSPA1A* (HSP70) and *HSPB1* (HSP27), which stimulate the innate immune system [34–37], were significantly increased. We examined whether the induction of these genes was characteristic to Bz treatment. Lymph node cells from 10-week-old and 14-week-old MRL/lpr mice were stimulated with Bz and protein kinase inhibitor staurosporine (Sta), which was used as another apoptosis inducer. Incubation of lymph node cells with Bz significantly increased *HSPA1A* and *HSPB1* expression when compared with that in the control mice, whereas Sta did not affect the expression (Additional file 4).

Six hours after injection of vehicle (control, *n* = 4), Bz (*n* = 4), or Cyc (*n* = 3) drugs into the 14-week-old mice, mRNA from the spleens was analyzed with the SurePrint G3 Mouse GE 8 × 60 K Microarray (Agilent Technologies). Compared with the control mice, significantly increased (>2-fold) and decreased (<0.5-fold) genes were extracted using Gene Spring software. The top 20 increased genes are presented in Table 1.

## Discussion

Patients with SLE with high disease activity who are refractory to conventional treatments require novel therapies. The proteasome inhibitor Bz is a potential novel therapeutic agent for SLE. In this study, we showed that the disease activity of MRL/lpr mice progressed from 10 to 14 weeks of age and was significantly decreased by Bz treatment. However, Bz treatment also increased the mortality rate of mice with high disease activity status, in spite of its therapeutic effects. The mechanism mediating the high mortality and toxicities needs clarification for the safe clinical application of Bz in patients with SLE.

MRL/lpr mice with a higher disease activity showed a significant increase in the number of B cells, plasma cells, CD4^+^ T cells, CD8^+^ T cells, and lpr T cells in the spleen and bone marrow. The same was observed in the lymph nodes, except for the plasma cells (Fig. 1b). The major cell source for splenomegaly and lymphadenopathy was the accumulation of a large number of lpr T cells, which are double negative, polyclonal, and nonneoplastic [38]. The origin of lpr T cells remains unclear [39] and how lpr T cells functionally contribute to autoimmunity on the MRL background is also unknown. Bobe et al. [30] reported that arsenic trioxide exerted a selective cytotoxic effect on lpr T cells. Although the mechanism is unclear, lpr T cells with a defective intrinsic Fas-mediated apoptotic pathway might be sensitive to external cellular stress. MRL/lpr mice at 14 weeks of age were burdened with high lpr T cells that appear relatively vulnerable to Bz cytotoxic effects as well as plasma cells, including induction of secondary necrosis and apoptosis or necroptosis as described previously [40].

Consistent with previous reports [7–14], Bz had a therapeutic effect even in advanced lupus (14-week-old MRL/lpr mice), resulting in marked decreases in serum immunoglobulins and anti-dsDNA antibodies and improved glomerulonephritis. Several groups have reported improved survival rates in Bz-treated NZW/B [7, 9, 10, 13, 14] and MRL/lpr [7, 14] mice. However, these results for the survival rates were not consistent with the present study. The reason for this contradiction might be due to the differences in mouse age, disease activity, or just mouse strains. In our study, Bz treatment of MRL/lpr mice was initiated at 14 weeks of age, which is later than in previous reports [7, 14], designed to examine the ameliorative and not protective effects of Bz on disease activity, considering the clinical use. Because older (14-week-old) MRL/lpr mice harbor high lpr T cells, which Bz selectively targets (as well as plasma cells), the lethal and toxic effects of Bz may be due to the killing of a large number of lymphocytes. After discontinuation of Bz treatment, the mice survival rate clearly improved, although the anti-dsDNA antibody level resurged. As reported previously by Khodadadi et al. [10], maintenance therapy might be required for a complete immunological remission after Bz treatment. One reason for the improved mice survival rates compared with the continuously Bz-treated mice could be just the removal of toxicity. Another reason might be the therapeutic effect after the acute toxic phase shown in Fig. 6 because the survival rate after discontinuation of Bz treatment from 18 weeks of age was higher than the survival rate in the control mice (the survival rates from 18 weeks of age until 43 weeks of age were 83% vs 50%, respectively). This indicated that Bz treatment, at least for a limited duration, might provide survival advantages despite the resurgence of anti-dsDNA antibody.

Bz and Cyc treatments resulted in a significant change in the expression levels of a number of genes in splenocytes (Additional file 1). No genes or canonical pathways were commonly shared between Bz and Cyc treatments (Additional files 1 and 2), suggesting that the molecular responses to these treatments were totally different. IPA revealed that DNA damage-related pathways (GADD45 and DNA damage-induced 14-3-3 signaling) were most significantly associated with Cyc treatment (Additional file 2). This suggested that Cyc treatment, which alkylates DNA, induces DNA damage and activates the cell cycle checkpoint. IPA also showed that the protein ubiquitin pathway was most significantly associated with Bz treatment. The protein ubiquitin pathway is the process for the degradation of unfolded proteins [41]. The amount of HSP family and proteasome subunit proteins was significantly increased with Bz treatment (Additional file 3). HSPs function as molecular chaperons and unfold misfolded intracellular proteins to decrease ER stress [42]. Bz treatment may also activate the counteracting system against ER stress as well as ER stress in lymphocytes.

Bz has been reported to have several side effects on therapeutic uses [43], some of which may be related to cytokine levels [44, 45]. In an LPS-induced shock mouse, which is considered to be a model of sepsis, serum cytokines such as IL-6 and TNF-α were increased, and these cytokines induced organ injury and mortality [46–48]. In our study, cytoreduction by Bz may induce proinflammatory cytokine release at an early phase, and this inflammation could be lethal to some MRL/lpr mice at 14 weeks of age. At 18 weeks of age, serum IL-6 and TNF-α levels were rather decreased in surviving mice treated from both 10 and 14 weeks of age. High IL-6 and TNF-α levels may be associated with lethality in mice up to 18 weeks of age. It is also possible that Bz exerts anti-inflammatory effects by inhibiting NF-κB activation [49] after the clearance of the majority of Bz-targeting cells, such as plasma and lpr T cells. ER stress has been considered to trigger cell death via apoptosis [4, 50], without inducing inflammatory reactions. Programmed necrosis, “necroptosis,” which was originally reported as Fas-triggered and caspase-independent necrotic cell death, is mediated by receptor-interacting protein [51] and inhibited by Necrostatin [52]. In contrast to apoptosis, necroptosis or secondary necrosis is followed by innate immune inflammation via the release of damage-associated molecular patterns (DAMPs), such as HMBG1 and HSPs [53–55]. In addition to their function as molecular chaperones with a cellular protective effect, HSPs also function as DAMPs and have proinflammatory potential in immunogenic cell death [56–58], which might have lethal effects. As shown in Table 1 and Additional file 4, *HSPA1A* (HSP70) and *HSPB1* (HSP27) were characteristically induced with Bz treatment both in vivo and in vitro. Because our study was limited to the use of data obtained from the whole spleen or lymph node cells, it still remains unknown which cell subsets are the source of such DAMPs. Bz treatment resulted in higher amounts, although not significant, of *HSPA1A* in lymph node cells at 14 weeks of age when compared with treatment at 10 weeks of age, as shown in Additional file 4. This might represent the higher frequency of Bz-sensitive lpr T cells in 14-week-old mice (Fig. 2b). Bz might cause immunogenic lymphocyte death associated with the induction of DAMPs, such as HSPs, followed by stimulation of the innate immune system and increased inflammatory cytokine release. Indeed, the IL-6 level was significantly increased (3.135-fold) in the splenocytes of Bz-treated mice but not of Cyc-treated mice (Additional file 2), which supports our idea.

MRL/lpr mice may have some lymphoproliferative disorder even at younger age. Because Bz induced massive cell death, the death of mice might be due to tumor lysis syndrome which is defined as a complication of metabolic abnormalities caused by the rapid release of intracellular components. Following massive tumor cell death, the diagnostic criteria include hyperuricemia, hypocalcemia, hyperphosphatemia, and renal failure [59]. After treatment with Bz, however, serum uric acid, calcium, and phosphate levels did not significantly change between Bz-untreated and Bz-treated MRL/lpr mice at 14 weeks of age (Additional file 5). We conclude that the main cause of the death of mice in this case is not tumor lysis syndrome but systemic inflammation. Although there are no defined counterparts for lpr T cells in SLE patients, plasmablasts, which are defined as CD19^lo^CD27^++^ or CD27^++^CD38^+^ cells that can secrete immunoglobulin molecules, were reported to be increased in number in the peripheral blood of SLE patients [60, 61]. Alexander et al. [16] reported that Bz significantly reduced plasmablasts in the peripheral blood. Active SLE patients with increased Bz-sensitive plasmablasts are more likely to develop inflammation-associated complications than inactive SLE patients.

Because HSP70 [34–36] and HSP27 [37] were reported to activate the innate immune system via Toll-like receptor 4 (TLR4), we designed dexamethasone (Dex) administration to Bz-treated mice expecting an inhibitory effect on lower survival. As reported in LPS model mice [62, 63], Dex would counteract with acute phase reactions induced by Bz; however, this failed to prevent the death of Bz-treated mice (Additional file 6). We assumed that Dex may have been ineffective in counteracting acute phase reactions due to its short biological half-life. In the experiment depicted in Additional file 6, Dex was injected only before Bz injection, twice a week (72-h or 96-h intervals). The half-life of Dex seemed to be just a few hours in rats [64, 65]. One possible reason for the high mortality rate despite Dex administration may be because the anti-inflammatory effect waned off during the intervals between doses. After cessation of the anti-inflammatory effect of Dex, DAMP release may restimulate the innate immune system and have some rebound effect on inflammation associated with steroid withdrawal. To control the acute systemic inflammation after Bz administration, we may need to consider more frequent administration of dexamethasone or a more specific strategy, such as anti-IL-6 receptor therapy [66].

## Conclusions

In summary, Bz, which could target plasma cells to mediate its therapeutic effects in lupus-like disease, exerted lethal toxic effects in MRL/lpr mice with high disease activity. Systemic inflammatory reaction triggered by cell death from Bz treatment might be lethal to MRL/lpr mice. Clarification of the molecular mechanisms of the effects of Bz is needed prior to its application as a preventive strategy in human SLE. We propose that it is important to take the disease activity of these mice models into consideration when elucidating the therapeutic and toxic effects of this novel drug for SLE.

## Additional files

Additional file 1: is a table presenting significantly upregulated and downregulated genes in spleens of Bz-treated and Cyc-treated mice. Six hours after injection of 14-week-old MRL/lpr mice with vehicle (control, *n* = 4), Bz (*n* = 4), or Cyc (*n* = 3), mRNA of spleens was analyzed with SurePrint G3 Mouse GE 8 × 60 K Microarray (Agilent Technologies). Compared with control mice, significantly increased (>2-fold) and decreased (<0.5-fold) genes were extracted using Gene Spring software applying a modified *t* test. (XLSX 24 kb)

Additional file 2: is a figure showing the top significantly regulated (*p* < 0.05) canonical pathways as assessed by IPA of Bz and Cyc regulated genes. Significantly upregulated and downregulated genes after treatment with Bz and Cyc, which are indicated in Additional file 1, were analyzed with IPA for canonical pathways. (TIF 26624 kb)

Additional file 3: is a table presenting significantly increased or decreased genes among the proteasome ubiquitin pathway, GADD45 signaling, and DNA damage induced 14-3-3 signaling. The genes which were significantly increased with Bz treatment and belong to the gene set of the proteasome ubiquitin pathway, and the genes which were significantly increased with Cyc treatment and belong to the gene set of GADD45 signaling and DNA damage induced 14-3-3 signaling were extracted with IPA analysis. (XLSX 10 kb)

Additional file 4: is a figure showing in-vitro induction of *HSPA1A* (HSP70) and *HSPB1* (HSP27) expression in Bz-treated lymph node cells. Lymph node cells from 10-week-old and 14-week-old MRL/lpr mice (*n* = 3 each) were treated with Bz (100 μM) and staurosporine (Sta, 100 μM). After 6 hours of treatment, cDNA was synthesized and gene expression of *HSPA1A*, *HSPB1*, and *GAPDH* was measured using quantitative PCR. Ratio values of copy numbers of *HSPA1A* and *HSPB1* to *GAPDH* genes expressed as mean number ± SEM. Statistical analysis performed using one-way ANOVA analyses with Turkey post-hoc test. (TIF 33139 kb)

Additional file 5: is a figure showing the effect of Bz treatment on serum uric acid, calcium, and phosphate. Mice aged 14 weeks were injected with vehicle or Bz, twice on both day 1 and day 4 (*14wk* and *14wkBz*, respectively). After the second Bz injection, the mice were sacrificed, and blood specimens were analyzed for uric acid, calcium, and phosphate levels. Values expressed as median with quartile range. (TIF 8887 kb)

Additional file 6: is a figure showing the effects of coadministration of dexamathasone (*Dex*) on the lethal and toxic effects of Bz. Fourteen-week-old MRL/lpr mice were subcutaneously injected with Bz (750 μg/kg, twice a week) with (*blue*, *Bz + Dex*) or without (*red*, *Bz*) injection of Dex (10 μg/body). Coadministration of Dex did not prevent the lethal toxic effects of Bz. (TIF 8900 kb)

## Abbreviations

ALT: Alanine aminotransferase
anti-dsDNA: Anti-double-stranded DNA
AST: Aspartate aminotransferase
BSA: Bovine serum albumin
Bz: Bortezomib
Cr: Creatinine
Cyc: Cyclophosphamide
DAMP: Damage associated molecular protein
Dex: Dexamethasone
ER: Endoplasmic reticulum
GADD45: Growth arrest and DNA damage inducible 45
GAPDH: Glyceraldehyde-3-phosphate dehydrogenase
HSP: Heat shock protein
IFN-γ: Interferon gamma
IgG: Immunoglobulin G
IgM: Immunoglobulin M
IL-6: Interleukin-6
PBS: Phosphate buffered saline
RBC: Red blood cell
SCID: Severe combined immunodeficiency disease
SLE: Systemic lupus erythematosus
Sta: Staurosporine
TNF-α: Tumor necrosis factor alpha
WBC: White blood cell
