# Supplementary figures and images for: Bortezomib treatment induces a higher mortality rate in lupus model mice with a higher disease activity
Source: Arthritis Res Ther. 2017 Aug 11;19:187. doi: 10.1186/s13075-017-1397-7 (PMC5553803; doi:10.1186/s13075-017-1397-7)

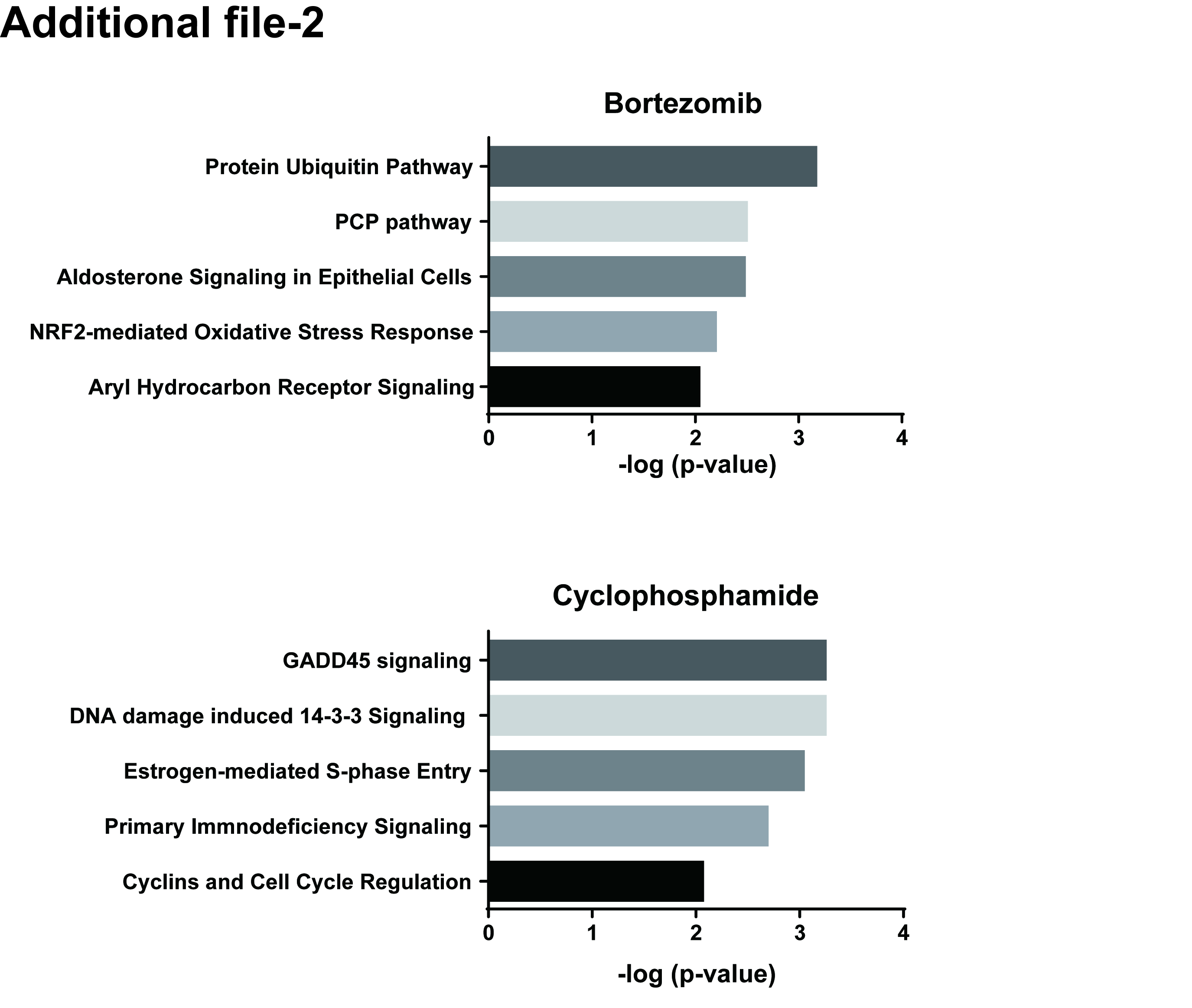

Supplement: Supplementary file 2 — is a figure showing the top significantly regulated (p < 0.05) canonical pathways as assessed by IPA of Bz and Cyc regulated genes. Significantly upregulated and downregulated genes after treatment with Bz and Cyc, which are indicated in Additional file 1, were analyzed with IPA for canonical pathways. (TIF 26624 kb) [file 13075_2017_1397_MOESM2_ESM.tif]

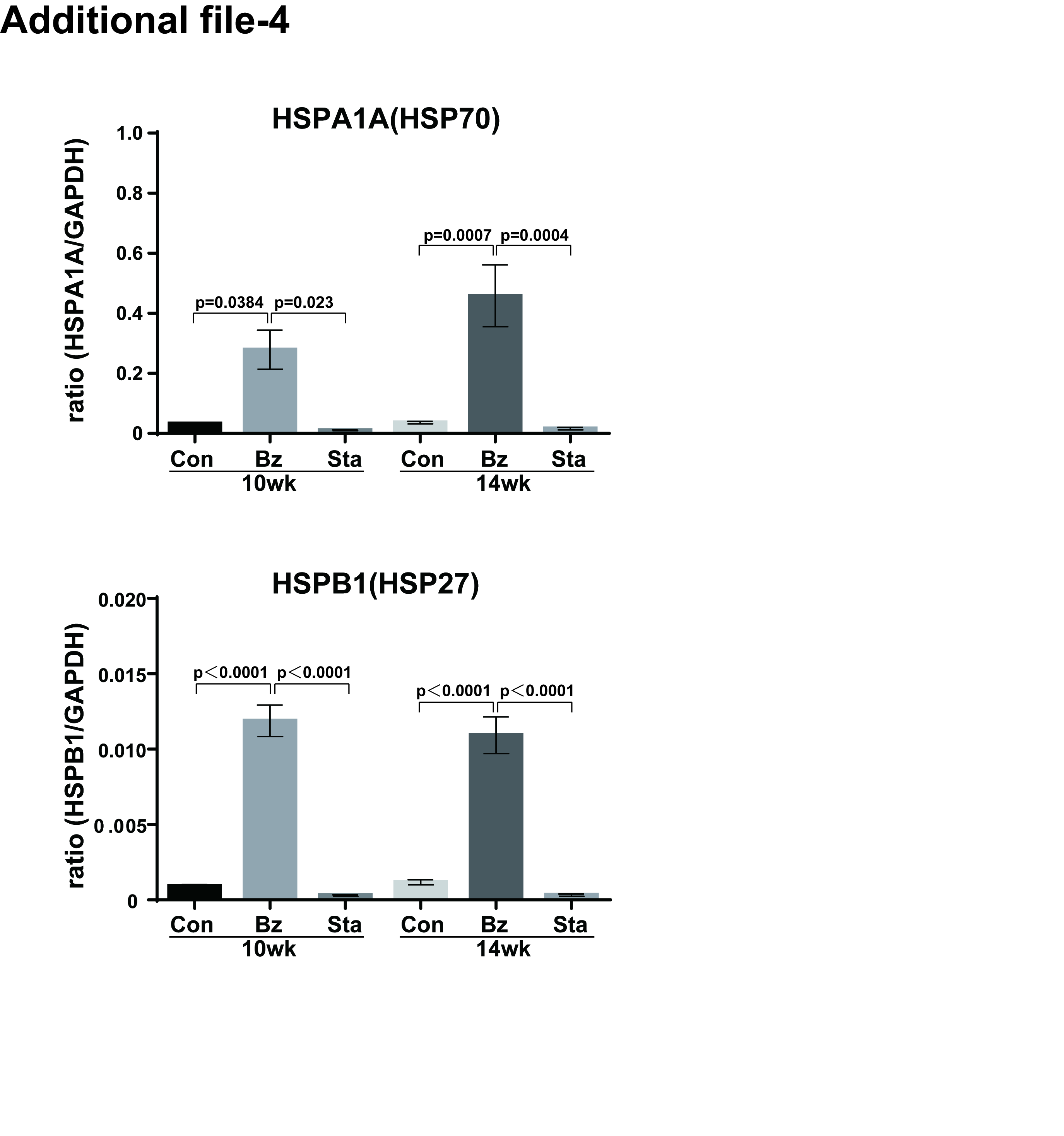

Supplement: Supplementary file 4 — is a figure showing in-vitro induction of HSPA1A (HSP70) and HSPB1 (HSP27) expression in Bz-treated lymph node cells. Lymph node cells from 10-week-old and 14-week-old MRL/lpr mice (n = 3 each) were treated with Bz (100 μM) and staurosporine (Sta, 100 μM). After 6 hours of treatment, cDNA was synthesized and gene expression of HSPA1A, HSPB1, and GAPDH was measured using quantitative PCR. Ratio values of copy numbers of HSPA1A and HSPB1 to GAPDH genes expressed as mean number ± SEM. Statistical analysis performed using one-way ANOVA analyses with Turkey post-hoc test. (TIF 33139 kb) [file 13075_2017_1397_MOESM4_ESM.tif]

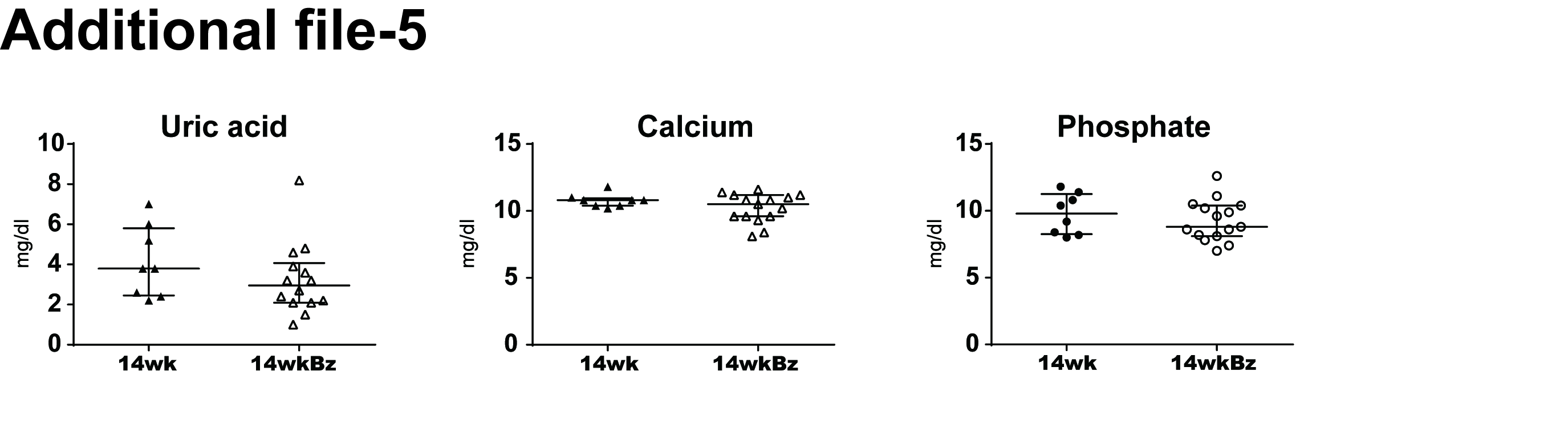

Supplement: Supplementary file 5 — is a figure showing the effect of Bz treatment on serum uric acid, calcium, and phosphate. Mice aged 14 weeks were injected with vehicle or Bz, twice on both day 1 and day 4 (14wk and 14wkBz, respectively). After the second Bz injection, the mice were sacrificed, and blood specimens were analyzed for uric acid, calcium, and phosphate levels. Values expressed as median with quartile range. (TIF 8887 kb) [file 13075_2017_1397_MOESM5_ESM.tif]

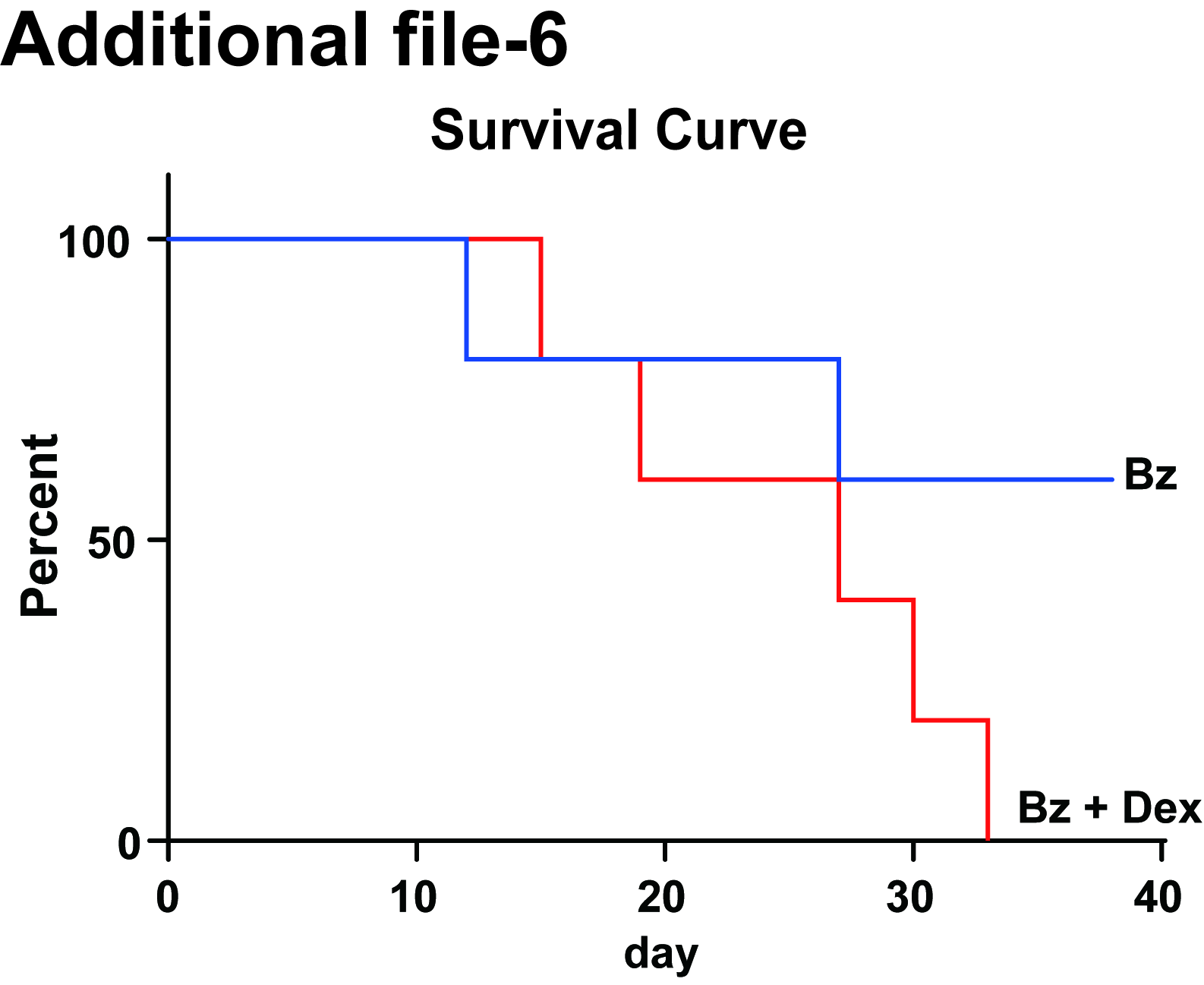

Supplement: Supplementary file 6 — is a figure showing the effects of coadministration of dexamathasone (Dex) on the lethal and toxic effects of Bz. Fourteen-week-old MRL/lpr mice were subcutaneously injected with Bz (750 μg/kg, twice a week) with (blue, Bz + Dex) or without (red, Bz) injection of Dex (10 μg/body). Coadministration of Dex did not prevent the lethal toxic effects of Bz. (TIF 8900 kb) [file 13075_2017_1397_MOESM6_ESM.tif]
